# Organizational Support and Job Satisfaction of Nurses in Contemporary Clinical Practice: Indirect Associations Through Distributive Justice and Emotional Exhaustion

**DOI:** 10.3390/healthcare14172750

**Published:** 2026-08-28

**Authors:** Marin Mamić, Nikolina Farčić, Slavka Galić, Vesna Ćosić, Ivana Mamić, Ivana Barać, Zvjezdana Gvozdanović, Goranka Rafaj, Silvija Marić, Ivan Vukoja, Robert Lovrić

**Affiliations:** 1 General County Hospital Požega, Osječka 107, 34000 Požega, Croatia; mmamic@fdmz.hr (M.M.); ivana.mamic@pozeska-bolnica.hr (I.M.); ibarac@fdmz.hr (I.B.); silvija.maric@pozeska-bolnica.hr (S.M.); 2 Faculty of Dental Medicine and Health Osijek, Josip Juraj Strossmayer University of Osijek, 31000 Osijek, Croatia; vcosic@fdmz.hr (V.Ć.); zvjezdana.gvozdanovic@obnasice.hr (Z.G.); rlovric@fdmz.hr (R.L.); 3 Department of Neurosurgery, University Hospital Centre Osijek, 31000 Osijek, Croatia; 4 Department of Nursing, University of Applied Sciences in Bjelovar, 43000 Bjelovar, Croatia; grafaj@vub.hr; 5 Department of Social Sciences and Humanities, University of Slavonski Brod, 35000 Slavonski Brod, Croatia; slavka.galic@pozeska-bolnica.hr; 6 General County Hospital Našice, 31500 Našice, Croatia; 7 Faculty of Medicine, Josip Juraj Strossmayer University of Osijek, 31000 Osijek, Croatia; 8 Faculty of Medicine, University of Rijeka, Ul. Braće Branchetta 20/1, 51000 Rijeka, Croatia

**Keywords:** nurses, organizational support, distributive justice, emotional exhaustion, job satisfaction, burnout, structural equation modeling

## Abstract

**Background/Objectives:** Nurses’ job satisfaction is influenced by organizational conditions and psychological responses to demanding work. Although perceived organizational support, organizational justice, and burnout are established correlates of nurses’ work outcomes, less is known about how distributive justice and emotional exhaustion may operate simultaneously within the association between organizational support and job satisfaction. This study examined these two theoretically distinct indirect pathways. **Methods:** A cross-sectional study was conducted among 157 nurses employed at General County Hospital Požega, Croatia, between November 2023 and January 2025. Perceived organizational support, distributive justice, emotional exhaustion, and job satisfaction were assessed using standardized questionnaires. Structural equation modeling used robust maximum likelihood estimation with full information maximum likelihood for partially missing data; indirect associations were evaluated in a separate maximum-likelihood model using 5000 bootstrap samples. **Results:** Greater perceived organizational support was associated with more favorable perceptions of distributive justice and lower emotional exhaustion. Greater distributive justice was associated with higher job satisfaction, whereas emotional exhaustion was associated with lower job satisfaction. The remaining direct association between organizational support and job satisfaction was not statistically significant after both pathways were included. Bootstrap analyses supported indirect associations through both distributive justice and emotional exhaustion. A sensitivity analysis showed that reversing the ordering of distributive justice and organizational support yielded identical global fit indices. **Conclusions:** Organizational support, perceived fairness, and emotional exhaustion appear to be closely interconnected aspects of nurses’ work experience. The findings integrate organizational and psychological perspectives and identify supportive management, fair resource allocation, and the prevention of emotional exhaustion as complementary organizational targets. Because of the cross-sectional design, the pathways should be interpreted as statistical indirect associations rather than causal mechanisms.

## 1. Introduction

Nurses’ job satisfaction is an important indicator of professional well-being, workforce stability, and the functioning and quality of healthcare organizations. It is associated with organizational commitment, intention to remain in the organization, job performance, and the quality of healthcare provided [1,2]. In hospital settings, nurses work under conditions characterized by continuous responsibility for patients, emotionally demanding situations, shift work, time pressure, and frequently limited staffing and material resources. Their job satisfaction is therefore shaped not only by individual characteristics but also by the organizational context in which care is delivered [3,4].

The quality of the work environment is consistently associated with nurses’ professional and organizational outcomes. Supportive leadership, adequate organizational resources, professional autonomy, and participation in decision-making have been associated with greater job satisfaction and more favorable workforce outcomes [5,6,7]. In contrast, excessive workload, insufficient resources, limited autonomy, and inadequate organizational support may contribute to emotional exhaustion, lower job satisfaction, and greater intentions to leave [8]. Understanding the organizational conditions associated with nurses’ job satisfaction is therefore important not only for employee well-being but also for healthcare workforce sustainability and the quality of care.

Perceived organizational support, organizational justice, and burnout have each received considerable attention in nursing and organizational research. Perceived organizational support has been associated with job satisfaction, professional well-being, burnout, and turnover-related outcomes [9,10,11,12,13,14]. Organizational justice, including distributive justice, has similarly been associated with job satisfaction and burnout [15,16,17,18,19], while emotional exhaustion represents an important psychological response to prolonged occupational demands [20].

Nevertheless, these constructs are frequently examined separately or in models focusing on individual relationships, leaving limited evidence on how organizational evaluations and psychological strain may operate simultaneously in relation to nurses’ job satisfaction.

An important unresolved issue concerns the mechanisms through which perceived organizational support is associated with job satisfaction. In particular, distributive justice and emotional exhaustion may represent two conceptually different processes: an organizational evaluative process concerning the perceived fairness of outcomes and a psychological process involving the depletion of emotional resources. Examining these processes simultaneously may provide a more differentiated understanding of how nurses’ perceptions of their organization are associated with their attitudes toward their work.

The present study therefore integrates Organizational Support Theory (OST), Social Exchange Theory (SET), and the Job Demands–Resources (JD-R) Model to examine the relationships among perceived organizational support, distributive justice, emotional exhaustion, and job satisfaction. Each framework serves a distinct theoretical function: OST explains the formation and relevance of employees’ general perceptions of organizational support; SET provides a broader explanation of reciprocal employee–organization relationships and employees’ evaluations of organizational treatment; and the JD-R Model explains how organizational resources may be associated with psychological strain and emotional exhaustion.

The primary aim of this study was to examine the association between perceived organizational support and nurses’ job satisfaction and to determine whether distributive justice and emotional exhaustion represent two parallel indirect pathways that may statistically account for this association. By examining these organizational and psychological processes simultaneously, this study sought to contribute to a more integrated understanding of nurses’ job satisfaction and to identify organizational conditions that may be relevant to nursing management and workforce well-being.

### 1.1. Theoretical Background and Hypothesis Development

#### 1.1.1. Perceived Organizational Support and Job Satisfaction

Organizational Support Theory proposes that employees develop a general perception concerning the extent to which their organization values their contributions and cares about their well-being [9]. These perceptions are formed through employees’ interpretations of organizational policies, managerial decisions, rewards, working conditions, and the treatment they receive from organizational representatives [10,11]. Because employees tend to attribute such actions to the organization itself, favorable organizational treatment may contribute to a generalized perception that the organization values and supports them.

Social Exchange Theory complements this perspective by conceptualizing employee–organization relationships as reciprocal exchanges [15]. When employees perceive that the organization values their contributions, responds to their needs, and provides adequate support and resources, they may reciprocate with more favorable attitudes toward their work and organization. Job satisfaction may therefore represent one important attitudinal correlate of perceived organizational support.

Meta-analytic evidence has demonstrated consistent positive associations between perceived organizational support and favorable employee outcomes, including job satisfaction, organizational commitment, affective well-being, and performance [10,11]. Similar relationships have been reported among nurses, with higher perceived organizational support associated with greater job satisfaction and more favorable professional outcomes [2,12,13,14]. On this theoretical and empirical basis, the following hypothesis was proposed:

**H1.**  *Perceived organizational support is positively associated with nurses’ job satisfaction.*

#### 1.1.2. Perceived Organizational Support and Distributive Justice

Organizational justice refers to employees’ perceptions of fairness within organizational processes, interactions, and outcomes [16]. Distributive justice specifically concerns the perceived fairness of the outcomes employees receive in relation to their contributions, responsibilities, and investments [16]. In nursing practice, these evaluations may concern the allocation of workload, shifts, responsibilities, recognition, rewards, opportunities for professional development, and access to organizational resources.

Perceived organizational support and distributive justice are therefore related but conceptually distinct. Perceived organizational support represents a broad evaluation of whether the organization values and supports the employee, whereas distributive justice concerns a more specific evaluation of whether organizational outcomes are distributed fairly [9,10,11,16].

The direction of the relationship between organizational support and organizational justice requires particular caution. Organizational Support Theory and the broader organizational behavior literature commonly conceptualize fair organizational treatment as an antecedent of perceived organizational support, because employees may interpret fair procedures and outcomes as evidence that the organization values their contributions and cares about their well-being [10,11,21]. Contemporary organizational justice research likewise emphasizes social-exchange mechanisms as a central relational process linking fairness evaluations with employees’ broader relationship with the organization [22]. Direct empirical evidence also supports this ordering: both procedural and distributive justice have been shown to contribute to the development of perceived organizational support [23], and distributive justice has been identified as an antecedent of perceived organizational support in an independent two-sample study [24]. Accordingly, an ordering in which distributive justice precedes perceived organizational support is theoretically and empirically well established and must be considered a plausible alternative.

However, the two constructs may also be related within a broader reciprocal employee–organization evaluation process. From an OST and SET perspective, employees’ generalized perception that the organization supports and values them may provide a broader interpretative context within which specific organizational decisions and outcomes are evaluated [9,10,11,15]. Employees who perceive stronger organizational support may consequently evaluate the allocation of organizational outcomes more favorably. In this interpretation, perceived organizational support represents a broad relational assessment, whereas distributive justice represents a more specific cognitive evaluation of organizational outcomes.

The present study specified perceived organizational support as preceding distributive justice in the primary conceptual model because it was conceptualized as the broader organizational perception and distributive justice as a more specific evaluation of organizational resource and outcome allocation. Importantly, this specification represents a theory-driven analytical ordering rather than a claim of established temporal or causal precedence. Because both constructs were measured cross-sectionally, the reverse ordering remains theoretically plausible and cannot be excluded empirically.

Within the primary theoretical specification used in this study, perceived organizational support was expected to be positively associated with distributive justice. This expectation reflects the proposed broad-to-specific evaluative ordering in which a general perception that the organization values and supports the employee provides an interpretative context for evaluating the fairness of specific organizational outcomes. This ordering is theory-driven and does not imply established temporal precedence.

**H2.**  *Perceived organizational support is positively associated with distributive justice.*

Distributive justice has also been consistently associated with favorable work attitudes. Employees who perceive workload, responsibilities, recognition, rewards, and organizational resources as being allocated fairly are more likely to evaluate their work positively. In nursing, organizational justice has been associated with job satisfaction and burnout [17,18], and previous research among Croatian nurses has shown positive associations between distributive and procedural justice and job satisfaction [19]. On this basis, the following hypothesis was proposed:

**H3.**  *Distributive justice is positively associated with nurses’ job satisfaction.*

If perceived organizational support is associated with more favorable evaluations of distributive justice, and distributive justice is in turn associated with greater job satisfaction, distributive justice may statistically account for part of the association between organizational support and job satisfaction. This proposed indirect pathway represents an organizational evaluative process rather than evidence of a causal or temporal mechanism.

**H4.**  *Perceived organizational support is indirectly and positively associated with nurses’ job satisfaction through distributive justice.*

#### 1.1.3. Perceived Organizational Support, Emotional Exhaustion, and Job Satisfaction

Emotional exhaustion represents a central dimension of occupational burnout and reflects the depletion of emotional and psychological resources resulting from prolonged exposure to work demands [20]. Nurses may be particularly susceptible to emotional exhaustion because their work involves continuous responsibility for patients, exposure to suffering and death, shift work, time pressure, interpersonal demands, and potentially insufficient staffing and opportunities for recovery [8].

The Job Demands–Resources Model provides the principal theoretical basis for the emotional exhaustion pathway examined in the present study. The JD-R Model distinguishes between job demands, which require sustained physical or psychological effort and may contribute to strain, and job resources, which facilitate goal attainment, reduce the impact of demands, and support employee motivation and well-being [25]. Workload, emotional demands, role conflict, and time pressure may operate as job demands, whereas managerial support, autonomy, access to information and resources, opportunities for development, and broader organizational support may function as job resources.

Within this framework, perceived organizational support can be understood as an organizational resource. Nurses who perceive that their organization recognizes their contributions, responds to their needs, and provides appropriate support and resources may be better equipped to cope with demanding working conditions. Conversely, insufficient organizational support may leave employees with fewer resources for managing sustained occupational demands and may therefore be associated with greater emotional exhaustion. Empirical studies among nurses have reported negative associations between perceived organizational support and burnout or emotional exhaustion [13,14,26].

Accordingly, the following hypothesis was proposed:

**H5.**  *Perceived organizational support is negatively associated with nurses’ emotional exhaustion.*

Emotional exhaustion may, in turn, be associated with nurses’ evaluations of their work. When emotional resources are depleted, employees may experience reduced capacity to cope with job demands and may develop less favorable attitudes toward their work. Previous research and systematic reviews have associated nurse burnout with lower job satisfaction, greater turnover intention, and adverse professional and organizational outcomes [8,27].

**H6.**  *Emotional exhaustion is negatively associated with nurses’ job satisfaction.*

Taken together, the JD-R framework suggests an indirect pathway in which organizational support is associated with lower emotional exhaustion and lower emotional exhaustion is associated with greater job satisfaction. In contrast to the distributive justice pathway, which represents an evaluation of organizational fairness, this pathway represents a psychological-energy process involving the preservation or depletion of employees’ emotional resources.

**H7.**  *Perceived organizational support is indirectly and positively associated with nurses’ job satisfaction through lower emotional exhaustion.*

#### 1.1.4. Parallel Indirect Pathways and the Conceptual Model

The proposed model integrates the preceding theoretical arguments by treating distributive justice and emotional exhaustion as conceptually distinct parallel pathways. Distributive justice represents a cognitive-evaluative organizational pathway concerning the fairness of organizational outcomes, whereas emotional exhaustion represents a psychological-energy pathway concerning the depletion of emotional resources.

A parallel rather than serial model was selected because the theoretical framework did not require distributive justice to precede emotional exhaustion or emotional exhaustion to precede distributive justice. Instead, both were hypothesized to represent independent pathways within the association between perceived organizational support and job satisfaction. This specification enables the two pathways to be estimated simultaneously without imposing an unsupported temporal ordering between the mediators.

**Figure 1:**
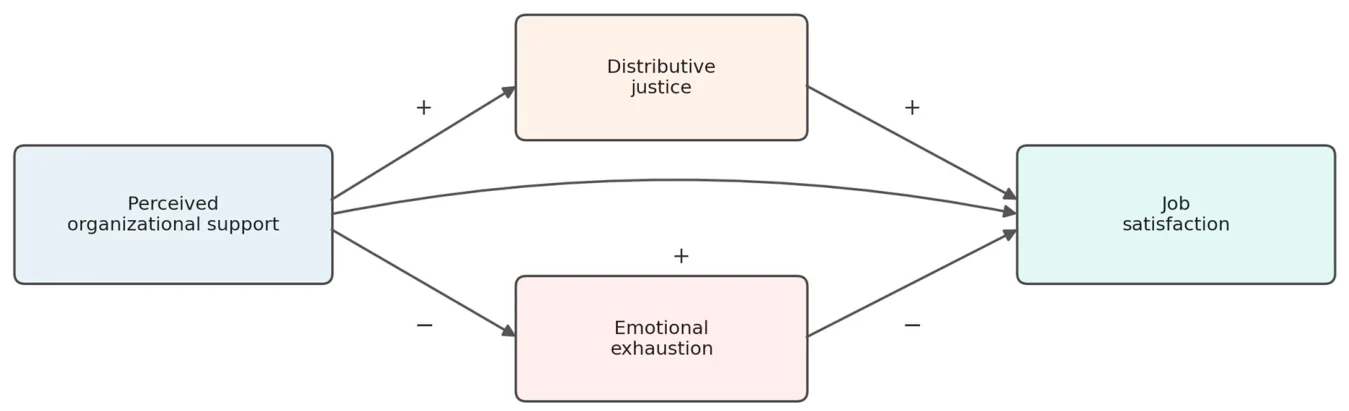
Hypothesized conceptual model of the two parallel indirect pathways linking perceived organizational support with job satisfaction. Note: The upper pathway represents the organizational evaluative pathway through distributive justice; the lower pathway represents the psychological-energy pathway through emotional exhaustion. The direct association between perceived organizational support and job satisfaction is estimated simultaneously. The arrows represent theory-informed statistical ordering and should not be interpreted as evidence of temporal or causal precedence.

This approach is consistent with the use of parallel multiple-mediator models to estimate specific indirect associations through multiple mediating variables while accounting for the other pathways included in the model [28]. Nevertheless, because the present study is cross-sectional, the estimated indirect effects should be interpreted as statistical indirect associations compatible with the proposed theoretical model rather than evidence of causal mediation. The hypothesized conceptual model is presented in Figure 1.

**H8.**  *Perceived organizational support is indirectly associated with nurses’ job satisfaction through two simultaneous parallel pathways: higher distributive justice and lower emotional exhaustion.*

The resulting conceptual model therefore integrates three complementary theoretical perspectives. OST provides the basis for understanding perceived organizational support as a broad employee evaluation of the organization [9,10,11]; SET explains how organizational treatment, support, fairness, and employee attitudes may be embedded within reciprocal employee–organization relationships [15]; and the JD-R Model specifically explains the pathway from organizational support through emotional exhaustion to job satisfaction [25]. Rather than using these theories interchangeably, the present model assigns each framework a distinct explanatory role.

Because previous literature provides a strong theoretical basis for considering fairness as an antecedent of perceived organizational support [10,11,21], and contemporary organizational justice research continues to emphasize social-exchange processes in employees’ responses to fairness [22], the proposed ordering in the primary model should not be interpreted as the only theoretically plausible specification. Accordingly, an alternative model in which distributive justice preceded perceived organizational support was additionally examined as a sensitivity analysis. This analysis was intended to assess the robustness of the proposed conceptual ordering and not to establish temporal precedence from cross-sectional data.

## 2. Materials and Methods

### 2.1. Study Design and Setting

A cross-sectional observational study was conducted to examine the associations among perceived organizational support, distributive justice, emotional exhaustion, and job satisfaction among nurses and to test the theoretically specified parallel indirect pathways. This study was conducted at General County Hospital Požega, Croatia, between November 2023 and January 2025.

General County Hospital Požega is a secondary-level healthcare institution providing inpatient and outpatient services across several clinical and diagnostic–therapeutic units. Approximately 230 nurses with different levels of nursing education were employed during the data-collection period. Nursing work was organized through shift-based schedules, with differences among units in patient characteristics, workload intensity, work organization, and staffing arrangements. Because unit-level nurse-to-patient ratios, patient acuity indicators, overtime hours, and other objective workload measures were not systematically collected for the present study, these contextual characteristics could not be included as covariates or examined separately. Accordingly, the findings refer to the overall nursing workforce of the institution rather than to comparisons among individual hospital units.

Although data collection extended from November 2023 to January 2025, neither the research protocol nor the instruments used were changed during this period. According to the available institutional information, there were no major changes in the hospital’s management structure, overall work organization, departmental organization, staffing structure, or methods of healthcare delivery. However, these organizational indicators were not systematically recorded longitudinally for the purposes of the present study. Consequently, smaller temporal variations in staffing, workload, or ward-level working conditions could not be formally assessed or statistically controlled.

This study was conducted in accordance with the Declaration of Helsinki and applicable ethical principles for research involving human participants. Approval was obtained from the Ethics Committee of General County Hospital Požega before data collection commenced (protocol code 02-7-3-3/1-2-2021; 27 August 2021). Participation was voluntary and anonymous, and informed consent was obtained from all participants.

### 2.2. Participants and Sample Size

The study population consisted of nurses and nursing technicians employed at General County Hospital Požega. Convenience sampling was used. During the study period, approximately 230 nurses employed at the hospital met the basic eligibility criteria, of whom 157 participated, corresponding to an approximate response rate of 68.3%.

The inclusion criteria were employment as a nurse or nursing technician at the hospital during the study period, age of at least 18 years, sufficient understanding of the Croatian language to complete the questionnaire independently, and voluntary consent to participate. Individuals who did not meet these criteria were not eligible to participate. Questionnaires were excluded from a specific analysis only when all information required to calculate the relevant study variable was missing. Participants with partially missing values on one or more variables included in the structural model were retained, with the available information incorporated through full information maximum likelihood estimation.

Because this study sought to include all eligible nurses available during the data-collection period, no a priori model-based sample-size calculation was performed. The achieved sample size was determined by the number of eligible nurses who agreed to participate and was subsequently evaluated using post hoc power and sensitivity analyses. To provide a transparent assessment of the statistical sensitivity of the available sample, post hoc power and sensitivity analyses for bivariate correlations were performed using G*Power version 3.1.9.7 (Heinrich Heine University Düsseldorf, Düsseldorf, Germany), with a two-tailed test, α = 0.05, and N = 151. For the smallest statistically significant correlation observed in this study (ρ = 0.209), the estimated achieved power was 0.73. For the remaining statistically significant correlations, ranging from ρ = 0.303 to ρ = 0.500, estimated power ranged from 0.97 to >0.99. Sensitivity analysis indicated that N = 151 permitted detection of correlations of approximately |ρ| = 0.226 with 80% power and |ρ| = 0.260 with 90% power. Thus, the available sample provided adequate sensitivity to detect small-to-moderate and larger bivariate associations, while sensitivity for very small associations was more limited. These calculations concern bivariate associations and do not constitute a formal power analysis of the indirect effects estimated within the structural equation model; small indirect associations should therefore be interpreted cautiously.

### 2.3. Measures

This study examined four principal constructs included in the structural model: perceived organizational support, distributive justice, emotional exhaustion, and job satisfaction. Basic sociodemographic characteristics, including sex, age, and educational level, were also collected.

No instrument was newly translated for the present study. The Croatian-language versions of the Organizational Justice Scale and the Job Satisfaction Index had previously undergone translation, cultural adaptation, and psychometric evaluation, with the relevant procedures and psychometric evidence reported in previous doctoral research [29]. These instruments were subsequently used in research conducted among nurses in Croatia [19]. The Croatian wording of the Survey of Perceived Organizational Support was obtained from research materials made available by the Department of Psychology, Faculty of Humanities and Social Sciences, University of Zagreb [9,30]. The emotional exhaustion subscale of the Maslach Burnout Inventory was administered in Croatian in accordance with the content and scoring instructions of the original instrument [20,31].

Perceived organizational support was assessed using the eight-item version of the Survey of Perceived Organizational Support (SPOS) [9]. Participants rated their agreement with each statement on a seven-point scale ranging from 1 (strongly disagree) to 7 (strongly agree). Negatively worded items were reverse-scored before calculating the total score, with higher scores indicating greater perceived organizational support. Internal consistency in the present sample was excellent (Cronbach’s α = 0.904).

Distributive justice was assessed using the four-item distributive justice subscale of the Organizational Justice Scale [16]. The items assess employees’ perceptions of the fairness of organizational outcomes, including the allocation of responsibilities, workload, rewards, and organizational resources. Responses were provided on a five-point scale, with higher scores indicating more favorable perceptions of distributive justice. Internal consistency in the present sample was excellent (Cronbach’s α = 0.933).

Job satisfaction was assessed using the five-item Job Satisfaction Index [29]. Participants responded using a five-point scale, and two negatively worded items were reverse-scored before calculation of the total score. Higher scores indicated greater job satisfaction. Internal consistency in the present sample was good (Cronbach’s α = 0.813).

Emotional exhaustion was assessed using the nine-item emotional exhaustion subscale of the Maslach Burnout Inventory [20,31]. The items assess feelings of emotional overload and exhaustion associated with professional demands. Responses were provided on a seven-point frequency scale ranging from 0 (never) to 6 (every day), with higher total scores indicating greater emotional exhaustion. Internal consistency in the present sample was good (Cronbach’s α = 0.881).

Cronbach’s α coefficients were calculated in the present sample for all four measures because the extent of formal Croatian validation evidence varied across instruments. The coefficients ranged from 0.813 to 0.933, indicating good-to-excellent internal consistency.

### 2.4. Data Collection Procedure

Data were collected between November 2023 and January 2025 using anonymous paper-and-pencil questionnaires completed independently by participants. Depending on the organization of work within individual departments, questionnaires were completed during working hours or immediately after the end of a shift. Researchers were not present during questionnaire completion in a manner that could influence participants’ responses.

Before participation, nurses received verbal and written information about the purpose of this study, the voluntary nature of participation, the anonymity of the collected data, and their right to withdraw without consequences. Completion of the questionnaire required approximately 15–20 min.

Questionnaires contained no personally identifying information. Participants were not subsequently contacted to provide missing responses. Missing values were not individually imputed; partially missing data used in the structural model were handled using FIML.

### 2.5. Statistical Analysis

Statistical analyses were performed using R version 4.5.2 (R Foundation for Statistical Computing, Vienna, Austria) [32]. Structural equation modeling was performed using the lavaan package [33].

Distributions of the principal study variables were initially examined using the Shapiro–Wilk test. Because three of the four principal variables showed statistically significant deviations from normality, continuous study variables were summarized using the median and interquartile range (Q1–Q3), while categorical variables were summarized using absolute and relative frequencies. Associations among the principal study variables were examined using Spearman’s rank correlation coefficient (ρ).

Structural equation modeling (SEM) was used to test the theoretically specified primary model. Perceived organizational support was specified as the exogenous variable, job satisfaction as the outcome, and distributive justice and emotional exhaustion as parallel intermediate variables. This specification reflected the a priori theoretical model developed from OST, SET, and the JD-R framework. The model estimated the direct association between perceived organizational support and job satisfaction, the specific indirect associations through distributive justice and emotional exhaustion, the total indirect association, and the total association. Because the design was cross-sectional, these pathways were interpreted as statistical associations compatible with the hypothesized model rather than evidence of temporal or causal mediation.

The primary structural model was estimated using robust maximum likelihood estimation (MLR), with the Yuan–Bentler correction of the χ^2^ statistic and robust Huber–White standard errors [34,35]. Partially missing data were handled using full information maximum likelihood (FIML), enabling all available observations to contribute to estimation without individual imputation [36]. Four participants with missing values for all four variables included in the structural model provided no information for model estimation and were therefore excluded, resulting in an analytic SEM sample of 153 participants. The individual principal variables contained between 151 and 153 valid observations.

Model fit was evaluated using the scaled χ^2^ statistic, comparative fit index (CFI), Tucker–Lewis index (TLI), root mean square error of approximation (RMSEA) with its 90% confidence interval, and standardized root mean square residual (SRMR). Because universal cutoff criteria for fit indices should not be applied mechanically, and because the specified model had only one degree of freedom, the fit indices were interpreted jointly and with particular caution [37,38]. Consequently, conclusions regarding the structural model were based on the theoretical specification and estimated pathways in addition to global fit statistics.

Direct path coefficients and their statistical significance were obtained from the MLR model. For the specific indirect, total indirect, and total associations, the same structural specification was separately estimated using maximum likelihood estimation with FIML and nonparametric bootstrapping with 5000 resamples. This separate ML model was used because the conventional nonparametric bootstrap procedure was not combined with the MLR estimator. Bootstrap standard errors and 95% percentile bootstrap confidence intervals were calculated for unstandardized indirect and total effects. An indirect association was considered statistically supported when its corresponding 95% bootstrap confidence interval did not include zero [28].

Given the theoretically plausible alternative ordering of perceived organizational support and distributive justice supported by previous organizational behavior research [10,11,21,22], an alternative model was examined as a sensitivity analysis. In this model, distributive justice was specified as preceding perceived organizational support, while retaining the theoretically supported pathway from perceived organizational support to emotional exhaustion and the associations of these constructs with job satisfaction. The primary and alternative models were estimated on the same data using identical MLR and FIML procedures. Their global fit indices and information criteria were compared descriptively. Because the models represented alternative theoretical orderings of cross-sectional associations, this sensitivity analysis was not interpreted as establishing causal or temporal precedence. Indirect associations in the alternative model were additionally evaluated using the same separate ML/FIML bootstrap procedure with 5000 resamples.

Statistical significance was set at *p* < 0.05.

## 3. Results

A total of 157 nurses participated in this study. Of these, 132 participants (84.1%) were women and 25 (15.9%) were men. Regarding educational level, 102 participants (65.0%) had completed secondary nursing education, 37 (23.5%) had a college-level qualification, and 18 (11.5%) had a university-level qualification. The median age of participants was 41 years (Q1–Q3 = 28–57).

Shapiro–Wilk tests indicated statistically significant deviations from normality for three of the four main study variables. Therefore, the main study variables were summarized using medians and interquartile ranges, and bivariate associations were examined using Spearman’s rank correlation coefficients.

### 3.1. Descriptive Statistics and Bivariate Associations

Descriptive statistics for the main study variables are presented in Table 1. The median organizational support score was 33 (Q1–Q3 = 26–39), while the median distributive justice score was 12 (Q1–Q3 = 8–15). The median emotional exhaustion score was 22 (Q1–Q3 = 14–30.5), and the median job satisfaction score was 19 (Q1–Q3 = 16–21). The number of valid observations ranged from 151 to 153 because of partially missing data.

Bivariate associations among the main study variables are presented in Table 2. Organizational support was positively correlated with distributive justice (ρ = 0.390, *p* < 0.001) and job satisfaction (ρ = 0.303, *p* < 0.001), and negatively correlated with emotional exhaustion (ρ = −0.482, *p* < 0.001). Job satisfaction was positively correlated with distributive justice (ρ = 0.311, *p* < 0.001) and negatively correlated with emotional exhaustion (ρ = −0.500, *p* < 0.001). Distributive justice was also negatively correlated with emotional exhaustion (ρ = −0.209, *p* = 0.010).

### 3.2. Primary Parallel Indirect-Effect Model

**Figure 2:**
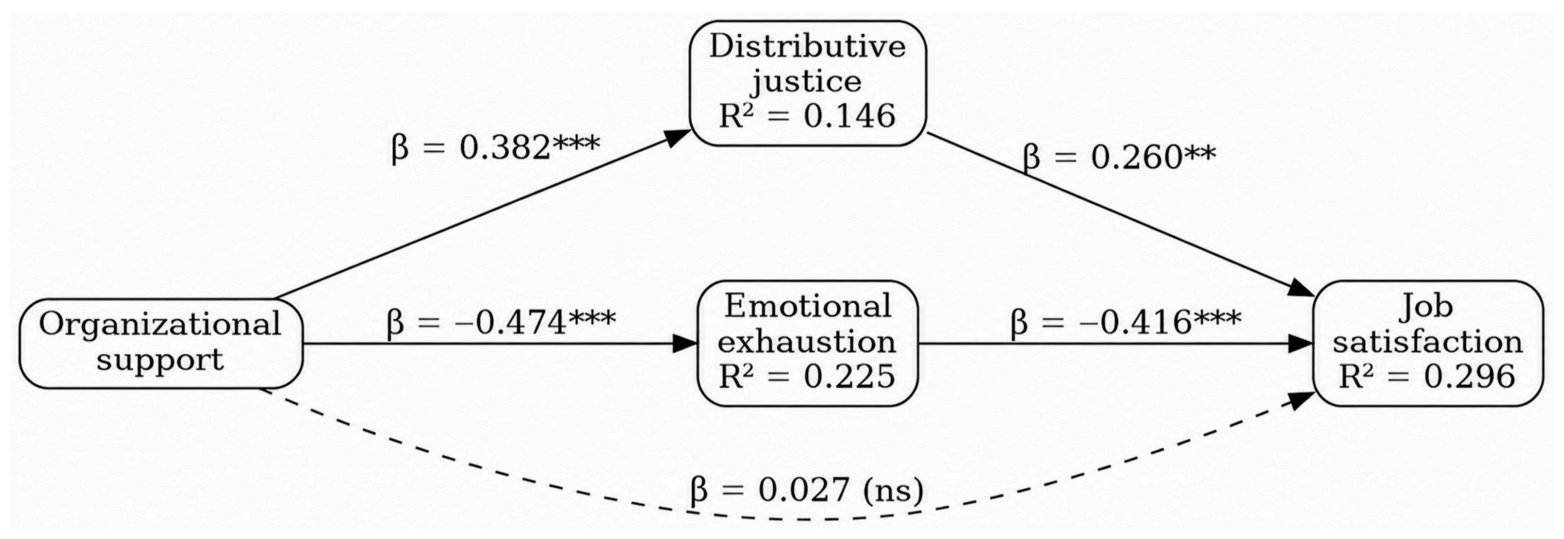
Estimated parallel indirect-effect model of the association between organizational support and job satisfaction through distributive justice and emotional exhaustion. Note: Solid lines indicate statistically significant associations, and the dashed line indicates the non-significant direct association. β—standardized regression coefficient; R^2^—explained variance; ns—not statistically significant. ** *p* < 0.01; *** *p* < 0.001.

Structural equation modeling was conducted to examine the theoretically specified association between perceived organizational support and nurses’ job satisfaction, with distributive justice and emotional exhaustion included as two parallel intermediate variables. Four participants had missing values for all four variables included in the structural model and therefore provided no information for model estimation, resulting in an analytic sample of 153 participants. Partially missing observations were retained using FIML. The primary model was estimated using MLR with the Yuan–Bentler correction and robust Huber–White standard errors. The estimated structural model is presented in Figure 2.

The global fit indices were numerically favorable: scaled χ^2^(1) = 0.175, *p* = 0.676, CFI = 1.000, TLI = 1.058, RMSEA = 0.000, 90% CI [0.000, 0.135], and SRMR = 0.009. However, because the model had only one degree of freedom, these indices provide limited evidence for distinguishing the specified model from plausible alternative models and should therefore be interpreted cautiously rather than as definitive confirmation of model adequacy (Table 3).

The primary model explained 14.6% of the variance in distributive justice, 22.5% of the variance in emotional exhaustion, and 29.6% of the variance in job satisfaction. The model therefore explained the largest proportion of variance in job satisfaction (Table 4).

Regression coefficients for the direct paths are presented in Table 5. Organizational support was positively associated with distributive justice (B = 0.159, β = 0.382, *p* < 0.001) and negatively associated with emotional exhaustion (B = −0.497, β = −0.474, *p* < 0.001). Distributive justice was positively associated with job satisfaction (B = 0.216, β = 0.260, *p* = 0.003), whereas emotional exhaustion was negatively associated with job satisfaction (B = −0.137, β = −0.416, *p* < 0.001). After distributive justice and emotional exhaustion were included in the model, the remaining direct association between organizational support and job satisfaction was not statistically significant (B = 0.009, β = 0.027, *p* = 0.765).

Indirect associations were estimated using 5000 bootstrap samples. The indirect association between organizational support and job satisfaction through distributive justice was statistically supported (B = 0.034, β = 0.099, 95% bootstrap CI [0.012, 0.058]). Thus, greater organizational support was associated with more favorable perceptions of distributive justice, which were in turn associated with greater job satisfaction.

The indirect association through emotional exhaustion was also statistically supported (B = 0.068, β = 0.197, 95% bootstrap CI [0.038, 0.103]). Greater organizational support was associated with lower emotional exhaustion, and lower emotional exhaustion was in turn associated with greater job satisfaction. The total indirect association was statistically supported (B = 0.102, β = 0.297, 95% bootstrap CI [0.069, 0.139]), as was the total association between organizational support and job satisfaction (B = 0.111, β = 0.323, 95% bootstrap CI [0.049, 0.171]).

Although the indirect association through emotional exhaustion was numerically larger than that through distributive justice, the difference between the two indirect associations was not statistically significant (B = −0.034, β = −0.098, *p* = 0.138, 95% bootstrap CI [−0.079, 0.010]). Therefore, the results do not support the conclusion that one of the two indirect pathways was significantly stronger than the other (Table 6).

H1 was supported at the bivariate level and for the total association between perceived organizational support and job satisfaction; however, the remaining direct association after inclusion of the two intermediate variables was not statistically significant. The associations specified in H2, H3, H5, and H6 were statistically significant and occurred in the hypothesized directions. The specific indirect associations proposed in H4 and H7 were supported by bootstrap confidence intervals that did not include zero, and the combined parallel indirect association proposed in H8 was also supported. The remaining direct path between organizational support and job satisfaction after inclusion of both parallel intermediate variables was not statistically significant.

### 3.3. Sensitivity Analysis: Alternative Theoretical Ordering

Because previous organizational behavior literature also supports a theoretically plausible ordering in which distributive justice precedes perceived organizational support, an alternative structural model reversing the ordering of these two constructs was examined as a sensitivity analysis. In this model, distributive justice was specified as associated with organizational support, organizational support was associated with emotional exhaustion, and distributive justice, organizational support, and emotional exhaustion were simultaneously associated with job satisfaction. The primary and alternative models were estimated using the same 153 observations and identical MLR/FIML procedures.

The alternative model produced the same global fit as the primary model. Both models yielded scaled χ^2^(1) = 0.175, *p* = 0.676, CFI = 1.000, TLI = 1.058, RMSEA = 0.000, 90% CI [0.000, 0.135], SRMR = 0.009, AIC = 3869.828, and BIC = 3909.224. Thus, global model fit provided no empirical basis for preferring one directional ordering of perceived organizational support and distributive justice over the other. The fit indices for the primary and alternative models are compared in Table 7.

Within the alternative model, distributive justice was positively associated with perceived organizational support (B = 0.921, β = 0.382, *p* < 0.001). Organizational support remained negatively associated with emotional exhaustion (B = −0.497, β = −0.474, *p* < 0.001), emotional exhaustion remained negatively associated with job satisfaction (B = −0.137, β = −0.416, *p* < 0.001), and distributive justice remained positively associated with job satisfaction (B = 0.216, β = 0.260, *p* = 0.003). The direct association between organizational support and job satisfaction remained non-significant (B = 0.009, β = 0.027, *p* = 0.765). Bootstrap analysis did not support the simple indirect association from distributive justice through organizational support to job satisfaction (B = 0.008, β = 0.010, 95% bootstrap CI [−0.042, 0.073]), but supported the sequential indirect association through organizational support and emotional exhaustion (B = 0.063, β = 0.075, 95% bootstrap CI [0.027, 0.110]).

The sensitivity analysis therefore showed that the cross-sectional covariance structure was equally compatible with both theoretically plausible orderings of perceived organizational support and distributive justice. The primary model was retained as the a priori theory-based specification, while the alternative model demonstrated that the present data cannot establish temporal precedence between these constructs.

## 4. Discussion

The present study examined perceived organizational support, distributive justice, emotional exhaustion, and job satisfaction within an integrated theoretical model among hospital nurses. The primary model distinguished two conceptually different indirect pathways: an organizational evaluative pathway involving distributive justice and a psychological-energy pathway involving emotional exhaustion. Both pathways were statistically supported. A prespecified sensitivity analysis addressing the theoretically plausible reverse ordering of distributive justice and organizational support produced identical global fit, underscoring that the cross-sectional data cannot determine temporal precedence between these closely related organizational perceptions.

The positive association between perceived organizational support and job satisfaction is consistent with Organizational Support Theory (OST), according to which employees form general beliefs regarding the extent to which their organization values their contribution and cares about their well-being [9,10,11]. Previous research has consistently associated stronger perceived organizational support with favorable employee attitudes, including greater job satisfaction and organizational commitment [10,11]. Similar associations have been reported among nurses, for whom organizational support has been related to greater job satisfaction, more favorable professional outcomes, and lower intentions to leave [2,12,13]. The present findings therefore reinforce the relevance of organizational support in nursing, but their principal contribution lies in examining how this broader organizational perception relates simultaneously to evaluations of fairness and psychological strain.

Social Exchange Theory (SET) provides a complementary interpretation of this pattern. Employee–organization relationships involve reciprocal evaluations of what employees contribute and what they receive from the organization [15]. Organizational recognition, access to resources, supportive management, and fair treatment may signal that the organization values the employee, while employees may respond with more favorable attitudes toward their work and organization. In this respect, perceived organizational support and distributive justice should not be considered interchangeable constructs. Organizational support represents a broad relational evaluation of the organization, whereas distributive justice concerns a more specific assessment of whether outcomes such as workload, responsibilities, recognition, rewards, and opportunities are distributed fairly [9,10,16].

The relationship between perceived organizational support and distributive justice deserves particular attention because it was the theoretically most ambiguous component of the proposed model. The primary model specified perceived organizational support as preceding distributive justice, based on the assumption that a general perception of being valued and supported by the organization may provide an interpretative context for employees’ evaluations of specific organizational decisions and outcomes. Under this interpretation, nurses who view their organization as supportive may also evaluate allocation practices more favorably. The observed positive association between the two constructs is compatible with this interpretation.

However, this is not the only theoretically plausible ordering. Organizational Support Theory also proposes that favorable organizational treatment contributes to the formation of perceived organizational support [9,10,11], and organizational justice research has repeatedly demonstrated that fairness evaluations are important components of employees’ responses to organizational practices [16,22]. Empirical studies have further shown that procedural and distributive justice can contribute to perceived organizational support [23] and that distributive justice may operate as an antecedent of perceived organizational support [24]. Thus, distributive justice may reasonably be conceptualized as an antecedent from which broader perceptions of organizational support are partly formed. This issue is particularly important because organizational justice has been associated with nurses’ job satisfaction, burnout, and intention to leave [17,18], while previous research among Croatian nurses has also shown positive associations between distributive and procedural justice and job satisfaction [19].

The sensitivity analysis directly addressed this theoretical ambiguity. Reversing the ordering between distributive justice and perceived organizational support produced identical global fit to the primary model. This equivalence should not be interpreted as evidence that the two theoretical explanations are equally true; rather, it shows that model fit cannot resolve their temporal ordering in cross-sectional data. Longitudinal, cross-lagged, or temporally separated designs are needed to determine whether fairness evaluations primarily precede broader support perceptions, the reverse occurs, or reciprocal processes operate over time.

This comparison clarifies the theoretical contribution of this study. The contribution is not a claim that organizational support causally determines distributive justice. Instead, this study integrates a broad relational perception of organizational support with a specific fairness evaluation and a psychological strain process within one model, while explicitly demonstrating the limits of directional inference from cross-sectional data.

The emotional exhaustion pathway is theoretically less ambiguous and is strongly aligned with the Job Demands–Resources (JD-R) Model [25]. The JD-R framework proposes that job resources can support employees in meeting occupational demands, maintaining motivation, and limiting the depletion of physical and psychological resources. Perceived organizational support may function as such a resource when nurses believe that their organization recognizes their contributions, responds to their needs, and provides resources and assistance when work demands increase. In contrast, insufficient organizational resources and support may leave employees more vulnerable to sustained psychological strain.

The negative association between organizational support and emotional exhaustion observed in this study is consistent with previous nursing research. Higher perceived organizational support has been associated with lower burnout among nurses in different healthcare settings [13,14,26]. This relationship may be particularly relevant in nursing because professional demands include not only workload and time pressure but also emotional exposure to suffering, responsibility for patient safety, shift work, and repeated interpersonal demands. Organizational support cannot eliminate these demands, but it may influence the resources available to nurses for managing them.

Emotional exhaustion was, in turn, associated with lower job satisfaction. This finding is consistent with the broader burnout literature and with evidence showing that nurse burnout is associated with poorer professional and organizational outcomes, including lower job satisfaction, greater turnover intention, and less favorable patient and organizational outcomes [8,27]. Within the JD-R framework, this relationship can be interpreted as a consequence of resource depletion: employees who experience sustained emotional exhaustion have fewer psychological resources available for maintaining positive involvement with their work.

The statistically supported indirect association through emotional exhaustion therefore provides a theoretically coherent extension of previous findings. Organizational support was related to lower emotional exhaustion, which was in turn related to greater job satisfaction. The importance of this pathway is not that it establishes a longitudinal mediation mechanism, which cannot be demonstrated with the present design, but that it connects a broad organizational resource with an important psychological state and an attitudinal work outcome within the same model. In this respect, the JD-R Model contributes something different from OST and SET: OST explains employees’ general perceptions of organizational support, SET provides a relational framework for understanding organizational treatment and work attitudes, whereas the JD-R Model specifically explains why organizational resources may be associated with lower emotional strain.

The parallel specification of distributive justice and emotional exhaustion also provides an additional conceptual contribution. Distributive justice captures a cognitive-evaluative aspect of nurses’ organizational experience, whereas emotional exhaustion represents a psychological-energy aspect. Both indirect pathways were supported in the primary model, and their magnitudes did not differ statistically. Therefore, the results do not justify treating one mechanism as more important than the other. Instead, they suggest that organizational evaluations and psychological well-being may represent complementary dimensions of nurses’ work experience. This distinction may help explain why interventions addressing only one component of the work environment may have limited effects if other important organizational or psychological conditions remain unfavorable.

The absence of a statistically significant residual direct association between perceived organizational support and job satisfaction following the inclusion of distributive justice and emotional exhaustion should also be interpreted cautiously. It does not imply that organizational support is unimportant for job satisfaction or that the indirect pathways represent proven causal mechanisms. Rather, within the specified cross-sectional model, the association between organizational support and job satisfaction was statistically accounted for to a substantial extent by the variables representing fairness evaluations and emotional exhaustion. This pattern is theoretically compatible with the proposed model, but alternative causal directions, reciprocal relationships, and unmeasured explanatory variables remain possible.

Taken together, the findings contribute to the organizational behavior and nursing literature in three respects. First, they integrate perceived organizational support, distributive justice, emotional exhaustion, and job satisfaction within a single theory-informed model rather than examining these relationships separately. Second, this study distinguishes an organizational evaluative pathway from a psychological-energy pathway and links each pathway to a different theoretical rationale. Third, by explicitly testing the theoretically plausible reverse ordering between distributive justice and perceived organizational support, this study demonstrates that the cross-sectional data cannot uniquely determine their temporal ordering. These findings therefore support theoretical integration while also emphasizing the need for caution when interpreting directional models derived from cross-sectional organizational data.

More specifically, the findings refine the theoretical literature by suggesting that perceived organizational support should not be treated as an isolated attitudinal predictor of job satisfaction. Its association with job satisfaction may be embedded in both employees’ cognitive evaluations of how organizational outcomes are distributed and their psychological capacity to cope with demanding work. The simultaneous consideration of evaluative and resource-depletion processes therefore provides a bridge between OST/SET and JD-R perspectives, which are often applied separately. For nursing research, this integrated interpretation broadens the explanatory focus from whether organizational support is associated with job satisfaction to how different dimensions of organizational experience may coexist within that association. Future longitudinal research should examine whether these processes unfold sequentially, reciprocally, or under specific organizational conditions.

### 4.1. Practical Implications

The findings have practical implications for nursing management, although they should not be interpreted as demonstrating that specific organizational interventions will necessarily cause improvements in job satisfaction. Instead, the observed pattern identifies organizational conditions that may represent relevant targets for management and future intervention studies.

Perceived organizational support may be strengthened through organizational practices that communicate recognition of employees’ contributions and responsiveness to their professional needs. These practices may include transparent communication, meaningful participation of nurses in decisions affecting their work, timely managerial feedback, access to professional development, recognition of professional contributions, and reliable managerial support when work demands increase. Such practices are consistent with the broader concept of organizational support and may contribute to a work environment in which nurses perceive that their professional role is valued [9,10,11,12].

The findings regarding distributive justice additionally indicate that support should not be expressed only through interpersonal communication or symbolic recognition. Nurses also evaluate concrete organizational outcomes. Hospital managers should therefore consider the perceived fairness of workload allocation, shift distribution, professional responsibilities, access to education and advancement opportunities, recognition, and other organizational resources. Transparent criteria for allocating such outcomes may be particularly important because even a generally supportive organizational climate may be evaluated less favorably when employees perceive that everyday outcomes are distributed inequitably [16,17,18,19].

Emotional exhaustion represents a complementary managerial concern. Strategies aimed at supporting nurses’ well-being may include adequate staffing relative to workload, opportunities for recovery, access to psychological support, early identification of occupational burnout, supportive supervision, and training of nurse managers to recognize and respond to excessive work demands. Such approaches are consistent with the JD-R perspective, which emphasizes the importance of strengthening job resources while reducing excessive demands [25]. Given the established relationship between nurse burnout and organizational and patient outcomes, prevention of emotional exhaustion should be considered relevant not only to employee well-being but also to workforce retention and healthcare quality [8,27].

Comparable organizational principles have been incorporated into international nursing initiatives. The American Nurses Credentialing Center Magnet Recognition Program emphasizes professional nursing leadership, shared decision-making, professional development, autonomy, and an organizational environment that supports high-quality nursing practice [39]. Studies comparing Magnet and non-Magnet hospitals have reported more favorable nurse and patient outcomes in Magnet environments [40,41]. Similarly, the American Association of Critical-Care Nurses’ Healthy Work Environment framework emphasizes skilled communication, true collaboration, effective decision-making, appropriate staffing, meaningful recognition, and authentic leadership [42]. These initiatives do not provide direct evidence that the specific pathways identified in the present study are causal, but they demonstrate that organizational support, fairness-related practices, staff participation, recognition, and working conditions are actionable components of contemporary nursing management.

The practical implication of the present findings is therefore not that hospitals should target job satisfaction as an isolated outcome. A more comprehensive approach would simultaneously strengthen supportive organizational resources, improve transparency and fairness in the allocation of organizational outcomes, and reduce conditions associated with emotional exhaustion. This integrated approach corresponds to the two conceptually distinct pathways identified in the primary model and may provide a more useful framework for developing and evaluating future organizational interventions.

For nurse managers, these findings can be translated into routine management practices. Unit-level managers can periodically review the distribution of shifts, overtime, patient assignments, leave, training opportunities, and recognition to identify patterns that staff may perceive as inequitable. Short, structured staff check-ins may help identify emerging workload and exhaustion concerns, while decisions affecting scheduling and resource allocation should be accompanied by clear criteria and explanations. At the organizational level, leadership teams can monitor staff feedback, absenteeism, overtime, turnover intention, and well-being indicators together rather than in isolation, using these data to identify units requiring additional managerial or staffing support. Such practices would allow supportive leadership, fairness, and the prevention of emotional exhaustion to be incorporated into routine workforce management rather than addressed only after dissatisfaction or burnout becomes pronounced.

### 4.2. Limitations and Directions for Future Research

Several limitations should be considered when interpreting the findings. The most important is the cross-sectional design. All principal variables were assessed within the same data-collection framework, and temporal precedence among perceived organizational support, distributive justice, emotional exhaustion, and job satisfaction cannot therefore be established. The estimated indirect effects represent statistical indirect associations compatible with the proposed theoretical model rather than evidence of causal mediation. Reverse and reciprocal associations are also plausible; for example, nurses with higher job satisfaction may evaluate organizational support and fairness more favorably.

The sensitivity analysis reinforces this limitation rather than eliminating it. The primary and alternative models yielded identical global fit indices, demonstrating that the available cross-sectional data could not discriminate between the specified ordering in which perceived organizational support precedes distributive justice and the theoretically plausible ordering in which distributive justice precedes perceived organizational support. Longitudinal designs with temporally separated measurements, cross-lagged approaches, or prospective intervention studies are therefore required to clarify the temporal relationships among these constructs.

All study variables were assessed using self-report instruments. Consequently, common method variance, social desirability, and general positive or negative response tendencies may have influenced the observed associations. Although the constructs were measured using established instruments with good to excellent internal consistency in the present sample, reliability does not eliminate potential shared-method bias. Future studies could combine self-reported perceptions with objective organizational indicators, managerial data, staffing measures, or longitudinal administrative outcomes.

This study was conducted in a single secondary-level hospital using convenience sampling, which limits generalizability to other hospitals, healthcare systems, professional contexts, and countries. Approximately 68.3% of eligible nurses participated, but systematic differences between participants and non-participants cannot be excluded. Multicenter studies including hospitals with different organizational structures, staffing models, geographical locations, and levels of healthcare provision would provide a stronger basis for assessing the generalizability of the proposed relationships.

Data collection also extended from November 2023 to January 2025. According to available institutional information, no major changes occurred in the overall management structure, organization of work, hospital departments, staffing structure, or healthcare delivery during this period. However, organizational indicators were not systematically monitored longitudinally for the purposes of this study. Smaller temporal changes in staffing, workload, or ward-level working conditions therefore cannot be completely excluded as potential influences on participants’ perceptions.

The achieved sample size was adequate for detecting small-to-moderate or larger bivariate associations according to the conducted sensitivity analysis, but the power assessment was based on bivariate correlations rather than on a simulation-based assessment of specific SEM indirect effects. Consequently, this study may have had limited sensitivity to very small indirect associations. Replication in larger samples would allow for more complex structural specifications, formal comparisons of alternative theoretical models, and more precise estimation of indirect associations.

Finally, the structural model had only one degree of freedom. Consequently, the very favorable CFI, TLI, RMSEA, and SRMR values should not be interpreted as strong evidence that the primary model represents a uniquely correct structural explanation. The identical global fit of the theoretically plausible alternative model directly illustrates this limitation. Conclusions should therefore rest primarily on theoretical reasoning, consistency with previous evidence, the estimated relationships, and replication in longitudinal data rather than on global fit indices alone.

Despite these limitations, this study adds to the existing literature by simultaneously examining organizational support, distributive justice, emotional exhaustion, and job satisfaction among nurses using a theory-informed structural framework and by formally examining a plausible alternative ordering of two closely related organizational constructs. The use of established measures, robust estimation, FIML for partially missing data, bootstrap confidence intervals for indirect associations, and an explicit sensitivity analysis strengthens the transparency of the analytical approach. These methodological features do not remove the limitations of a cross-sectional single-center study, but they provide a clearer basis for interpreting what the data do and do not support.

## 5. Conclusions

Perceived organizational support was associated with nurses’ job satisfaction within a broader pattern involving both organizational evaluations and psychological strain. In the primary theoretical model, distributive justice and emotional exhaustion represented two statistically supported parallel indirect pathways, suggesting that the association between organizational support and job satisfaction may be understood in relation to both perceptions of fairness and emotional well-being.

The sensitivity analysis showed that reversing the theoretical ordering between distributive justice and perceived organizational support yielded identical global fit indices. This does not replace the a priori primary model, but demonstrates that the present cross-sectional data cannot establish temporal precedence between these constructs. The reported indirect pathways should therefore be interpreted as theory-informed statistical associations rather than causal mediation.

By assigning distinct explanatory roles to Organizational Support Theory, Social Exchange Theory, and the Job Demands–Resources Model, this study provides an integrated theoretical framework for understanding these relationships. From a practical perspective, supportive management, transparent and fair allocation of organizational resources, appropriate staffing, meaningful recognition, and strategies aimed at preventing emotional exhaustion represent complementary priorities for nursing management and future organizational intervention research.

## Figures and Tables

**Table 1:** Descriptive Statistics for the Main Study Variables.

Variable	Valid	Missing	Mdn	Q1	Q3	Minimum	Maximum	W	*p*
Job satisfaction	153	4	19	16	21	7	25	0.962	<0.001
Emotional exhaustion	151	6	22	14	30.5	3	47	0.974	0.006
Organizational support	152	5	33	26	39	8	55	0.987	0.164
Distributive justice	152	5	12	8	15	4	20	0.955	<0.001

Note: Valid—number of participants with available data; Missing—number of participants with missing data; Mdn—median; Q1—first quartile; Q3—third quartile; W—Shapiro–Wilk test statistic; *p*—statistical significance.

**Table 2:** Spearman’s Correlations among the Main Study Variables.

Variable	1	2	3	4
1. Distributive justice	1			
2. Emotional exhaustion	−0.209 *	1		
3. Organizational support	0.390 ***	−0.482 ***	1	
4. Job satisfaction	0.311 ***	−0.500 ***	0.303 ***	1

Note: Values are Spearman’s rank correlation coefficients; pairwise deletion was used for bivariate correlations, resulting in pairwise sample sizes ranging from 151 to 152; ρ—Spearman’s rank correlation coefficient; *p*—statistical significance. * *p* < 0.05; *** *p* < 0.001.

**Table 3:** Model Fit Indices for the Primary Parallel Indirect-Effect Model.

Fit Index	Value
χ^2^	0.175
df	1
*p*	0.676
CFI	1.000
TLI	1.058
RMSEA	0.000
RMSEA 90% CI	0.000–0.135
RMSEA *p*-value	0.790
SRMR	0.009
AIC	3869.828
BIC	3909.224
SABIC	3868.078

Note: χ^2^—scaled chi-square test of model fit; df–degrees of freedom; *p*—statistical significance; CFI—comparative fit index; TLI—Tucker–Lewis index; RMSEA—root mean square error of approximation; CI—confidence interval; RMSEA *p*-value—*p*-value for the test of close fit, H_0_: RMSEA ≤ 0.05; SRMR—standardized root mean square residual; AIC—Akaike information criterion; BIC—Bayesian information criterion; SABIC—sample-size adjusted Bayesian information criterion. Fit indices are based on the scaled test statistic.

**Table 4:** Explained Variance in Endogenous Variables.

Variable	R^2^
Distributive justice	0.146
Emotional exhaustion	0.225
Job satisfaction	0.296

Note: R^2^—coefficient of determination.

**Table 5:** Regression Coefficients in the Primary Parallel Indirect-Effect Model.

Outcome	Predictor	Path	B	SE	β	z	*p*	95% CI for B
Job satisfaction	Organizational support	c′	0.009	0.031	0.027	0.299	0.765	−0.051–0.070
Job satisfaction	Distributive justice	b1	0.216	0.072	0.260	2.983	0.003	0.074–0.358
Job satisfaction	Emotional exhaustion	b2	−0.137	0.027	−0.416	−5.123	<0.001	−0.189–−0.084
Emotional exhaustion	Organizational support	a2	−0.497	0.072	−0.474	−6.950	<0.001	−0.637–−0.357
Distributive justice	Organizational support	a1	0.159	0.029	0.382	5.483	<0.001	0.102–0.215

Note: B—unstandardized regression coefficient; SE—robust standard error; β—standardized regression coefficient; z—z-value; *p*—statistical significance; CI—confidence interval; a1—path from organizational support to distributive justice; a2—path from organizational support to emotional exhaustion; b1—path from distributive justice to job satisfaction; b2—path from emotional exhaustion to job satisfaction; c′—direct association between organizational support and job satisfaction after including both parallel intermediate variables.

**Table 6:** Bootstrap Estimates of Indirect, Total Indirect, and Total Associations in the Primary Model.

Association	B	Bootstrap SE	β	z	*p*	95% Bootstrap CI
Indirect association through distributive justice	0.034	0.012	0.099	2.871	0.004	0.012–0.058
Indirect association through emotional exhaustion	0.068	0.017	0.197	4.047	<0.001	0.038–0.103
Total indirect association	0.102	0.018	0.297	5.612	<0.001	0.069–0.139
Total association	0.111	0.031	0.323	3.571	<0.001	0.049–0.171
Difference between indirect associations	−0.034	0.023	−0.098	−1.485	0.138	−0.079–0.010

Note: B—unstandardized effect estimate; Bootstrap SE—bootstrap standard error; β—standardized effect estimate; z—z-value; *p*—statistical significance; CI—percentile bootstrap confidence interval based on 5000 bootstrap samples. Confidence intervals refer to the unstandardized effects.

**Table 7:** Comparison of Fit Indices for the Primary and Alternative Models.

Fit Index	Primary Model	Alternative Model
χ^2^	0.175	0.175
df	1	1
*p*	0.676	0.676
CFI	1.000	1.000
TLI	1.058	1.058
RMSEA	0.000	0.000
RMSEA 90% CI	0.000–0.135	0.000–0.135
SRMR	0.009	0.009
AIC	3869.828	3869.828
BIC	3909.224	3909.224

Note: χ^2^—scaled chi-square test of model fit; df—degrees of freedom; *p*—statistical significance; CFI—comparative fit index; TLI—Tucker–Lewis index; RMSEA—root mean square error of approximation; CI—confidence interval; SRMR—standardized root mean square residual; AIC—Akaike information criterion; BIC—Bayesian information criterion.

## Data Availability

The data presented in this study are available on request from the corresponding author. The data are not publicly available due to privacy and ethical restrictions.
